# OP59 Exploring The Feasibility Of Generative Artificial Intelligence In Replicating Health Economic Models For Early-Phase Decision Support

**DOI:** 10.1017/S0266462325101177

**Published:** 2025-12-29

**Authors:** Sumeyye Samur, Jakob Langer, Emir Gursel, I. Fatih Yildirim, Turgay Ayer, Ipek Ozer Stillman, Jagpreet Chhatwal

## Abstract

**Introduction:**

Generative artificial intelligence (AI) holds promise in aiding development of health economic models. Our objective was to explore the feasibility of using generative AI to replicate health economic models based on previously published models.

**Methods:**

We replicated a Markov model of ulcerative colitis described in the literature using a two-step approach. First, we used Python for large language model interactions and utilized ValueGen.AI, a GPT-4-based platform with multi-agent pipelines (CrewAI, LangChain, and OpenAI libraries), to extract model structure and parameters from the source. These parameters were implemented in R’s heemod package to construct and run the Markov model. Next, we repeated the experiment using a more detailed technical report of the same model. We evaluated generative AI’s performance by comparing its conceptualization and parameterization with the original sources.

**Results:**

Using the publication, the generative AI platform effectively extracted costs and quality-of-life inputs linked to health states. However, for health states and transition probabilities, initial attempts were less successful due to limited descriptions in the text, resulting in misinterpretations and conflicting health states. Performance improved considerably when we used the detailed technical report, which offered clearer and more structured information. Generative AI could not successfully extract all transition probability calculations for the defined model cycle length based on the formulas provided in the documents.

**Conclusions:**

Generative AI holds significant potential for replicating previously published health economic models. However, challenges remain in capturing detailed model parameters, particularly when description of modeling approach lacks clarity and transparency. Improving standardized reporting practices within the health economics and outcomes research field is needed to enable generative AI to better support stakeholders during HTA processes.

